# Quality of life in caregivers of children and adolescents with Osteogenesis Imperfecta

**DOI:** 10.1186/s12955-015-0226-4

**Published:** 2015-04-01

**Authors:** Ana Paula Vanz, Têmis M Félix, Neusa Sica da Rocha, Ida V D Schwartz

**Affiliations:** Postgraduate Program in Medical Sciences, Universidade Federal do Rio Grande do Sul, Porto Alegre, Brazil; Medical Genetics Service, Hospital de Clínicas de Porto Alegre, Porto Alegre, Brazil; Postgraduate Program in Medical Sciences: Psychiatry, Universidade Federal do Rio Grande do Sul, Porto Alegre, Brazil; Department of Genetics, Universidade Federal do Rio Grande do Sul, Porto Alegre, Brazil

**Keywords:** Osteogenesis imperfecta, Quality of life, Caregivers, WHOQOL-BREF

## Abstract

**Background:**

Osteogenesis imperfecta (OI) is a group of genetic disorders of collagen biosynthesis, characterized by low bone density leading to fractures. Most patients exhibit functional impairment and require the aid of a caregiver. The aim of this study is to assess the quality of life (QoL) of caregivers of patients with OI.

**Methods:**

In this cross-sectional study, a convenience sampling strategy was used to enroll adult caregivers of children and adolescents with OI who attended a referral center in southern Brazil. The WHOQOL-BREF instrument was used to assess QoL.

**Results:**

Twenty-four caregivers of 27 patients (10 with type I, 4 with type III, and 13 with type IV OI) were included in the study. Eighteen caregivers were the patients’ mothers, two had OI, and 22 cared for only one patient. Mean WHOQOL-BREF scores were 14.59 for the physical health domain, 13.80 for the psychological domain, 15.19 for the social relationships domain, and 12.87 for the environmental domain; the mean total QoL score was 14.16. QoL scores did not differ significantly according to patients’ OI type or number of fractures. Economic status was not correlated significantly with QoL scores.

**Conclusions:**

QoL appears to be impaired in caregivers of patients with OI. Additional studies are required to confirm these findings and to ascertain which factors account for this phenomenon.

## Background

The World Health Organization has defined quality of life (QoL) as “an individual’s perception of their position in life in the context of the culture and value systems in which they live and in relation to their goals, expectations, standards and concerns”. The World Health Organization Quality of Life (WHOQOL) Group has developed several tools for QoL assessment in research settings, such as the WHOQOL-100 questionnaire and its abridged version, the WHOQOL-BREF questionnaire, which is designed to assess global QoL in four domains (physical health, psychological, environmental, and social relationships) [1].

QoL measures have emerged in recent decades as essential tools for assessment of the impacts of diseases (especially chronic conditions) and therapeutic interventions, joining traditional indicators such as mortality. QoL assessment is particularly essential in the context of disabling chronic conditions that force patients to modify their routines or prevent them from carrying out activities of daily living. Furthermore, patients living with chronic illness may be overwhelmed by physical, emotional, and financial uncertainties, social dilemmas, and costly expenditures, generating other chronic conditions that affect the whole family [2,3].

Osteogenesis imperfecta (OI) is a group of disorders caused by impairment of collagen biosynthesis and characterized by minimal traumatic fractures, dentinogenesis imperfecta, and hearing loss. The clinical features of OI represent a continuum ranging from perinatal lethality through severe skeletal deformity, mobility impairment, and very short stature to nearly asymptomatic manifestation with mild predisposition to fractures, normal stature, and normal lifespan [4]. The estimated incidence of OI is 1 in 10,000 live births, and autosomal dominant is the most common pattern of inheritance [5].

Several molecular studies identified 17 genes involved in the biosynthesis of collagen as causing OI, however the classification of OI is based on clinical and radiological features. A classification that remains in widespread use defines five distinct types of OI (types I–V). Type I OI is the mildest form of the disease; in the majority of cases, bone deformities are slight or absent and patients have characteristic blue sclerae. Type II OI, the most severe form of the disease, is incompatible with life, and death may occur in utero. Type III OI, the most severe disease form that is compatible with life, is characterized by progressive skeletal deformity and severe short stature. Type IV OI is the most clinically diverse form of the condition, with phenotypes ranging from mild to severe. Finally, OI type V is associated with callus formation of hyperplastic callus after corrective surgery or fracture, absence of dentinogenesis imperfecta (DI) and progressive calcification of the interosseous membrane between radius-ulna and/or tibia-fibula and dislocation of the radial head [6].

In OI, moderate to severe clinical manifestations lead to physical disability. The disablement process is the result of the impacts of chronic disease conditions on body system functioning, on the individual’s ability to perform basic human functions, and on the individual’s relationship with and within society. Four main factors lead to disablement: active pathology, physical impairment, functional limitation, and disability. The conceptual structure of disablement was adapted for OI in a model where active pathology was represented by a structural defect in collagen production or conformation, leading to impairments (skeletal disproportion and joint dysfunction), which, in turn, produce a functional limitation that leads to disability (makes the individual dependent on caregiver assistance) [7,8].

Children and adolescents with chronic diseases such as OI require continuous and often complex care, which poses a daily challenge to their caregivers. Furthermore, the provision of care and attention to a patient with OI may change the dynamics and routines of his or her family [8,9]. These sudden changes in the family environment are a source of conflict. Furthermore, mothers often assume the role of caregiver, adding to their existing family duties. Mothers are also burdened with managing and attempting to solve issues resulting from the disease. Chronic disease in children is a trigger of high stress levels in caregivers [10,11].

A recent qualitative study investigated how parents of children with severe and mild OI shape and manage their children’s condition over time. The authors found that parental responses to OI are constituted by parents’ feelings and actions, and identified four successive phases of response: initial reaction, acceptance, normalization, and “passing the baton”. Each stage affected subsequent stages and was influenced by the severity of OI, parents’ individual characteristics, their day-to-day experiences and the entourage [12].

To date, only one study has examined the QoL of caregivers of patients with OI. The authors observed that environmental domain scores were worse in patients with severe OI than in those with mild OI, despite the receipt of more support from appropriate institutions by families in the severe OI group than by those in the mild OI group [13]. Within this context, the objective of the present study was to assess QoL in caregivers of children and adolescents with OI.

## Materials and methods

The research ethics committee of the Hospital de Clínicas de Porto Alegre (HCPA) approved the protocol of this study (no. 110080). All participants provided written informed consent prior to study enrollment.

Data for this cross-sectional study were collected from August 2011 to August 2012 at HCPA, a university hospital in Southern Brazil. A convenience sampling strategy was used to recruit caregivers of children and adolescents with OI. Caregivers were defined as individuals who had direct contact with children or adolescents with OI (care recipients) and were in charge of coordinating and providing for the recipients’ basic needs; this category included caregivers with OI. Subjects were recruited from the outpatient clinic of the Reference Center for Osteogenesis Imperfecta Treatment which is affiliated with the HCPA’s Medical Genetics Service. Participants were invited to enroll in the study after attending routine outpatient appointments with their care recipients.

Clinical data regarding type of OI of the subject, treatment used and number of fractures during life time were recorded. The number of OI cases under care of the caregiver and the diagnosis of OI of the caregiver were also reported.

As so far, no instrument has been developed specifically to assess QoL in caregivers of individuals living with chronic illnesses, we used in this study the validated Brazilian Portuguese version of the WHOQOL-BREF, a generic instrument. The WHOQOL-BREF contain 26 questions distributed in four QOL domains – Physical, Psychological, Social, and Environmental. These domains aim to analyze physical capacity, psychological well-being, social relationships, and the environment where the individual is inserted. The answers for each domain are transformed in scores ranging from 4 to 20, with higher scores indicating better QoL [1,14].

Participants’ socioeconomic status was assessed using a questionnaire based on the Brazilian Association of Research Companies Economic Classification Criterion. This questionnaire yields a score that can be used to stratify the population into socioeconomic status ranges (A1, A2, B1, B2, C1, C2, D, and E), with “A” corresponding to the highest score and “E” to the lowest score [15].

Statistical analysis was conducted using SPSS 18.0 software. Ordinal quantitative variables were expressed as means and standard deviations. Student’s *t*-test was used to assess potential associations between the number of fractures sustained by care recipients (dichotomized as ≤10 or >10 fractures) and caregivers’ QoL domain scores. The Kruskal–Wallis test was used to analyze the distribution of domain scores according to care recipients’ OI type. Pearson correlation coefficients were used to test associations between socioeconomic status variables and QoL scores. The scores obtained at the different domains were compared to the mean scores of the normal population at validation of the instrument [14]. The significance level was set at 5% (*p* < 0.05) and 95% confidence intervals were used.

## Results

Twenty-four caregivers from 24 unrelated families were included in this study. Their mean age was 39 ± 9.1 years, and two caregivers had OI (types I and IV, respectively) but no major physical limitation. Eighteen (75%) caregivers were female and 16 of them were mothers of subject with OI. These 24 caregivers were responsible for the care of 27 patients with OI. Thirteen care recipients had type IV, 10 had type I, and four had type III OI (Table 1).

**Table 1:** **Characteristics of caregivers included in the study sample**

**Characteristic**	***n (%)***
Gender (*n* = 24)	
Female	18 (75)
Male	6 (25)
Educational attainment, years of schooling (mean ± SD; *n* = 24)	8.22 ± 4
Relationship to care recipient (*n* = 24)	
Mother	16 (66.6)
Father	6 (25)
Grandmother	1 (4.1)
Maternal Stepmother	1 (4.1)
Number of patients with OI in caregiver’s care	
1	22 (91.6)
2	1 (4.1)
3	1 (4.1)
OI type of care recipient (*n*=27)	
I	10 (37)
III	4 (16.8)
IV	13 (48.14)
Socioeconomic class (*n* = 24)	
B2	1 (4.1)
C1	3 (12.5)
C2	4 (16.7)
D	14 (58.3)
E	2 (8.3)

OI, osteogenesis imperfecta; SD, standard deviation.

WHOQOL-BREF scores were highest for the social relationships domain and lowest for the environmental domain (Table 2). The distribution of WHOQOL-BREF scores was not associated with the number of fractures sustained by care recipients (*t*-test: physical health, *p* = 0.67; psychological, *p* = 0.25; social, *p* = 0.94; environmental, *p* = 0.66; overall, *p* = 0.86), OI type, or caregivers’ socioeconomic status.

**Table 2:** **Quality of life of caregivers of children and adolescents with osteogenesis imperfecta compared with control samples**

**WHOQOL-BREF domain**	**Present sample (n = 24)**	**Control Brazilian population** [14] **(n = 50)**	***p***
Physical	14.9 ± 3.29	16.6 ± 2.1	0.002*
Psychological	13.8 ± 2.8	15.6 ± 2.1	0.003*
Social	15.2 ± 3.7	15.5 ± 2.6	0.117
Environmental	12.87 ± 2.9	14.0 ± 2.1	<0.004*

Data are presented as mean scores ± standard deviations. *p < 0.05, present sample *vs*. control sample.

All mean domain scores of our participants were significantly lower than those of healthy individuals from the original instrument validation sample (Table 2) [14]. These differences persisted after exclusion of the two caregivers with OI from analyses.

## Discussion

The diagnosis of a chronic illness in a child or adolescent constitutes a major challenge for the patient and his or her family, due to the burden of the condition and the impact of new routines imposed by continuous treatment. Adaptation to these changes requires the preparedness of all involved in the family environment, who must restructure their lives to deal with the disease and its implications [9,10].

Teams of professionals often focus on patients and regard caregivers as individuals who must always be ready and vigilant, rarely recognizing that caregivers can be overwhelmed with duties and information. Caregivers may require attention and support as they adapt to care recipients’ diagnoses [16].

Qualitative assessments of the QoL of caregivers of children and adolescents with cancer have identified conflicting situations experienced by caregivers and other factors that may impact QoL, including the sacrifice of routine activities (i.e., school or work), adjustment to new living conditions, family involvement, changes in marital dynamics, personal satisfaction, social support, and care recipients’ age [17,18]. Few studies, however, have addressed QoL in caregivers of patients with OI. A Polish study addressed this topic in a sample comprising the parents of 25 children with OI using the WHOQOL-BREF. In that study, 56% of respondents reported good global QoL and 8% had scores corresponding to poor QoL. Mean domain scores (scale, 4–20) were: physical health, 12.2 ± 1.2; psychological, 15.04 ± 2.2; environmental, 13.32 ± 2.0; and social relationships, 14.28 ± 1.5. Environmental domain scores were lower among the parents of children with severe (type III) OI than among those of children with mild (type I or IV) OI [13].

Our data suggest that the majority of caregivers of patients with OI are their mothers, in agreement with a previous study showing that mothers were caregivers of 80% of children living with chronic disease. Mothers are generally more involved than fathers in the disease management process; they usually act as chaperones during their children’s hospital visits and are more likely to interact with care management teams [19].

Our participants’ WHOQOL-BREF scores were lowest in the environmental domain, which assesses respondents’ financial resources, physical safety and security, home environment, opportunities to acquire new information and skills, participation in and opportunities for recreation/leisure, and physical environment (pollution, noise, traffic, climate), as well as the accessibility and quality of health and social care and transportation [1]. WHOQOL-BREF environmental domain scores were also lowest in a sample of 608 Brazilian adolescents aged 14–20 years and in the sample of healthy individuals used to validate this instrument in Brazil [20]. Psychological domain scores were also lower in our sample than in the general population [14]. A national sample in the United States was used to assess whether parents’ self-reported psychological distress was related to the consequences of their children’s chronic health conditions; the researchers found that parents of children with functional limitations were more distressed than were parents whose children experienced other types of consequence (or none) of their condition [21]. We believe that caregivers’ impaired QoL in the psychological and environmental domains in this sample is attributable to having children with OI. Physical domain items assessed sleep quality, impact of pain on daily activities, need for medical treatment to function, amount of energy for everyday life, ability to get around, and satisfaction with work. The needs of children and adolescents with OI, who require special developmental support and treatment, explain the low scores observed in these domains.

The absence of a significant association between OI type and QoL domain scores in the present study contrasts with the significant association observed between environmental domain scores and type III OI in a Polish sample [13]. This difference may be due to the small number of patients with type III OI in the present study.

Assessment of socioeconomic status in this sample showed that eight of the 24 (33%) caregivers were in class C1. Socioeconomic data based on a 2005 Brazilian Institute of Statistic and Public Opinion survey show that 20.7% of the Brazilian population belongs to the C1 class. Since 2005, improvement in the population’s economic status has been observed [22,23]. Economic level was not correlated with QoL scores in this study, in contrast to the positive correlations between socioeconomic level and environmental and social domain scores reported by a previous study preformed in Southern Brazil [24].

## Conclusion

In our study using WHOQOL-BREF, the caregivers of patients with OI had significant lower scores for the physical health, psychological, and environmental domains. Larger samples and more appropriate study designs are required to identify the factors involved in this QoL impairment. Thus, future investigations require a joint effort from all researchers in this field, to enable more reliable analysis of the cornerstones of caregivers’ QoL. Elucidation of these factors may enable direct interventions to address the major challenges faced by caregivers and, consequently, ensure comprehensive and efficient care of patients with OI.

## Abbreviations

OI: Osteogenesis Imperfecta
QoL: Quality of life
WHOQOL: World Health Organization quality of life
HCPA: Hospital de Clinicas de Porto Alegre
SD: Standard deviation
